# Apolipoprotein C3 and necrotic core volume are correlated but also associated with future cardiovascular events

**DOI:** 10.1038/s41598-022-18914-1

**Published:** 2022-08-25

**Authors:** Takayuki Ohwada, Takayuki Sakamoto, Satoshi Suzuki, Yukiko Sugawara, Kazuya Sakamoto, Ayano Ikeda, Fumika Haga, Tomoki Sato, Kazuhiko Nakazato, Yasuchika Takeishi, Kenichi Watanabe

**Affiliations:** 1 Department of Cardiology, Fukushima Red Cross Hospital, Fukushima, Japan; 2 Department of Cardiology, Takeda General Hospital, Aizuwakamatsu, Japan; 3 Department of Cardiovascular Medicine, Fukushima Medical University, Fukushima, Japan

**Keywords:** Biomarkers, Cardiology, Risk factors

## Abstract

We aimed to clarify the relationship between apolipoprotein C3 (apo-C3) and the vascular composition of lesion plaque in stable coronary disease (SCD) before percutaneous coronary intervention (PCI), and to investigate major adverse cardiovascular events (MACEs) within 4 years. Data of 98 consecutive patients with SCD who underwent PCI between November 1, 2012, and March 10, 2015, were analyzed. Laboratory and virtual histology-intravascular ultrasound (VH-IVUS) examinations of culprit lesions were conducted before PCI. Patients were divided according to median apo-C3 into low apo-C3 (≤ 8.5 mg/dL) and high apo-C3 (> 8.5 mg/dL) groups. VH-IVUS data indicated that the percentage of necrotic core volume (%NC) was significantly higher in the high apo-C3 group than in the low apo-C3 group. Moreover, the %NC significantly correlated with the apo-C3 level (R = 0.2109, *P* = 0.037). Kaplan–Meier curve analysis revealed that freedom from MACEs exhibited a greater decrease in the high apo-C3 group than in the low apo-C3 group, and in the high %NC group than in the low %NC group. Multivariate Cox hazards analysis showed that the %NC and high apo-C3 were independent predictors of 4 year MACEs. Apo-C3 may be a useful marker of future MACEs in patients with SCD after PCI and contribute to %NC growth.

## Introduction

In a meta-analysis^1^ and several studies^2,3^ that have investigated the plaque volume and vessel composition using virtual histology-intravascular ultrasound (VH-IVUS) during percutaneous coronary intervention (PCI) before and after taking statins, statins reduced the plaque volume^4^ and percentage of necrotic core volume (%NC). Previously, we showed the correlation of the %NC with apolipoprotein B and low-density lipoprotein (LDL) cholesterol^5^. These effects are presumed to be partly due to the lowering of LDL cholesterol. However, previous reports^6,7^ have shown that the cholesterol-lowering effect of strong statins on major adverse cardiovascular events (MACEs) was only 24% when compared with that of placebo. Thus, LDL cholesterol may not be the only factor that affects plaque volume and vessel composition.

Apolipoprotein C3 (apo-C3) is associated with triglyceride-rich lipoprotein metabolism and has emerged as an independent marker of the risk for cardiovascular disease (CVD)^8–13^. Apo-C3 concentrations in chylomicron-free serum were reported to be associated with event-free survival in patients with coronary artery disease^13^. Nonetheless, thus far, the relationship between apo-C3 and vessel composition has not been clarified. In particular, NC is a key feature of vulnerable plaque^14^, and MACEs associated with NC have not been investigated.

Therefore, in this study, we conducted VH-IVUS before PCI to examine the vessel structure and to evaluate the relationship between apo-C3 and vessel composition. We also analyzed the Kaplan–Meier curves for MACEs associated with apo-C3 or the %NC in the plaque in patients with stable coronary disease (SCD) after PCI.

## Results

### Comparisons of clinical characteristics, laboratory data, and IVUS data between high and low apo-C3 groups

During the follow-up period of 48 months, 26 MACEs occurred (one cardiac death, two acute myocardial infarctions, five unstable anginas, 21 new progressive lesions (including 15 lesions requiring PCI), 5 restenoses of the PCI site (including 4 lesions requiring PCI) and two arteriosclerosis obliterans requiring endovascular therapy (Supplementary Table S1). Patients were divided according to the median apo-C3 value into low apo-C3 (≤ 8.5 mg/dL) and high apo-C3 (> 8.5 mg/dL) groups (Tables 1, 2 and 3). Comparisons of clinical characteristics and oral medication between the groups are presented in Table 1. The body mass index was higher in the high apo-C3 group than in the low apo-C3 group.

**Table 1:** Comparisons of clinical characteristics and oral medications between the low and high apo-C3 groups.

	Low apo-C3 group (≤ 8.5 mg/dL, *n* = 52)	High apo-C3 group (> 8.5 mg/dL, *n* = 46)	*P*-value
Age (years)	67.9 ± 1.2	71.5 ± 1.4	0.0533
Male patients, no. (%)	40 (76.9)	34 (73.9)	0.7295
BMI	23.8 ± 0.5	25.7 ± 0.5	0.001*
**Clinical history**			
DM	15 (28.8)	17 (37.0)	0.3929
Hypertension	34 (65.4)	38 (82.6)	0.0539
Family history	6 (11.5)	9 (19.6)	0.2707
Smoking	15 (28.8)	17 (37.0)	0.3929
Previous PCI	23 (44.2)	15 (32.6)	0.2386
Previous MI	9 (17.3)	7 (15.2)	0.7799
**Oral medications**			
CCB	24 (46.2)	25 (54.3)	0.4181
ACEI/ARB	25 (48.1)	21 (45.7)	0.8103
Antiplatelet drugs	38 (73.1)	31 (67.4)	0.5383
Anti-DM drugs	9 (17.3)	6 (13.0)	0.5585
Fibrates	1 (1.9)	1 (2.2)	0.9302
Statin	22 (42.3)	23 (50.0)	0.4457

Values are presented as mean ± standard errors or as numbers (percentages) of patients.

*P < 0.05.

*apo-C3* apolipoprotein C3; *BMI* body mass index; *DM* diabetes mellitus; *PCI* percutaneous coronary intervention; *MI* myocardial infarction; *CCB* calcium-channel blocker; *ACEI/ARB* angiotensin-converting enzyme inhibitor/angiotensin II receptor blocker.

**Table 2:** Comparisons of laboratory data between the low and high apo-C3 groups.

	Low apo-C3 group (≤ 8.5 mg/dL, *n* = 52)	High apo-C3 group (> 8.5 mg/dL, *n* = 46)	*P*-value
**Laboratory data**			
TC (mg/dL)	174.8 ± 4.1	206 ± 6.0	< 0.001*
TG (mg/dL)	117.7 ± 6.4	210.6 ± 12.8	< 0.0011*
HDL-C (mg/dL)	52.9 ± 1.7	55.0 ± 1.9	0.4122
non-HDL-C (mg/dL)	121.9 ± 4.0	151.2 ± 6.2	< 0.001*
LDL-C (mg/dL)	109 ± 3.3	94.9 ± 5.8	0.0304*
Apo-A1 (mg/dL)	128.4 ± 2.7	138.4 ± 2.7	0.0107*
Apo-B (mg/dL)	83.8 ± 2.3	102.6 ± 3.9	< 0.001*
Apo-C3 (mg/dL)	7.0 ± 0.15	12.3 ± 0.50	< 0.001*
eGFR	68.1 ± 2.0	65.1 ± 2.2	0.32
HbA1c (%)	5.9 ± 0.101	6.3 ± 0.183	0.0292*
Hs-CRP (mg/dL)	0.154 ± 0.051	0.348 ± 0.071	0.0258*

Values are presented as mean ± standard errors. *P < 0.05.

*apo-C3* Apolipoprotein C3; *TC* Total cholesterol; *TG*, Triglyceride; *HDL-C* High-density lipoprotein cholesterol;* LDL-C* Low-density lipoprotein cholesterol; *eGFR* Estimated glomerular filtration rate; *HbA1c* hemoglobin A1c; *hs-CRP* High-sensitivity C-reactive protein.

**Table 3:** Comparisons of grayscale IVUS, VH-IVUS data, and MACEs between the low and high apo-C3 groups.

	Low apo-C3 group (≤ 8.5 mg/dL, *n* = 52)	High apo-C3 group (> 8.5 mg/dL, *n* = 46)	*P*-value
**Gray-scale data**			
Vessel volume (mm^3^)	789.0 ± 87.9	806.9 ± 66.1	0.8731
Plaque volume (mm^3^)	480.2 ± 35.4	316.7 ± 27.1	0.8868
Lumen volume (mm^3^)	308.8 ± 54.3	480.2 ± 54.3	0.8633
%Plaque burden (%)	60.9 ± 0.9	61.1 ± 1.3	0.8655
Length (mm)	54.8 ± 0.9	55.6 ± 0.8	0.5658
**VH-IVUS data**			
FI volume	179.7 ± 23.1	182.3 ± 18.9	0.9321
FF volume	84.5 ± 12.6	65.4 ± 6.9	0.2028
NC volume	48.2 ± 5.9	58.9 ± 8.3	0.2884
DC volume	15.8 ± 1.5	19.1 ± 2.4	0.2328
%FI	54.8 ± 0.9	55.0 ± 0.9	0.5658
%FF	24.1 ± 1.2	20.2 ± 1.0	0.0172*
%NC	15.0 ± 0.7	17.9 ± 0.7	0.0143*
%DC	6.1 ± 0.5	6.8 ± 0.5	0.5982
**MACEs**			
Total MACE (%)	7 (13.5)	19 (41.3)	0.0018*
AMI (%)	1 (1.9)	1 (2.2)	0.9302
UAP (%)	3 (5.8)	2 (4.3)	0.7496
Cardiac death (%)	0 (0)	1 (2.2)	0.2852
Restenosis (%)	2 (3.8)	3 (6.5)	0.5480
New lesion (%)	3 (5.8)	18 (39.1)	< 0.001*
EVT (%)	0 (0)	2 (4.3)	0.1287

Values are presented as mean ± standard errors or as numbers (percentages) of patients. *P < 0.05.

*apo-C3* Apolipoprotein C3; *IVUS* Intravascular ultrasound; *VH-IVUS* Virtual histology-intravascular ultrasound; *FI* Fibrous; *FF* FIbrofatty; *NC* Necrotic core; *DC* Dense calcium; *%FI* Percentage of the FI volume in the plaque volume; *%FF* Percentage of the FF volume in the plaque volume; *%NC* Percentage of the NC volume in the plaque volume; *%DC* Percentage of the DC volume in the plaque volume; *MACE* Major cardiovascular event (defined in the manuscript); *AMI* Acute myocardial infarction; *UAP* Unstable angina pectoris; *EVT* Endovascular therapy; *CI* Cerebral infarction.

**Figure 1:**
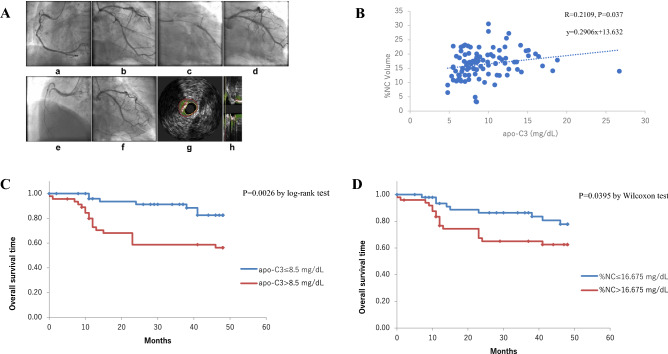
Representative case in the high apo-C3 group (**A**) Coronary angiograms of a representative case (a male patient) and virtual histology-intravascular ultrasound (VH-IVUS) of the lesion. (**a**) An intermediate lesion in the proximal right coronary artery at pre-percutaneous coronary intervention (PCI). (**b**) The target severe stenotic tandem lesion in the left circumflex coronary artery (LCX). (**c**) Two drug-eluting stents were implanted. (**d**) Coronary angiography findings after stenting. (**e**, **f**) Eight months later, the patient experienced acute myocardial infarction and was transported to our hospital by ambulance; coronary angiography was immediately performed. (**e**) The mid-right coronary artery, which had an intermediate lesion before PCI, was completely occluded. (**f**) The LCX showed no restenosis. (**g**) The VH-IVUS cross-sectional image of the lesion that underwent PCI in the LCX. (**h**) The longitudinal VH-IVUS image of the same lesion. In the color image, green indicates the fibrous region; yellowish green, the fibrofatty region; red, the necrotic core; and white, dense calcium. In this patient, the percentage necrotic core volume was 22.43%, and the apolipoprotein C3 level was 8.8 mg/dL. (**B**) Correlation curve between apo-C3 and %NC volume. There was a significant correlation between apo-C3 and %NC volume (*P* = 0.037). (**C**) Kaplan–Meier curves between patients with high apo-C3 and those with low apo-C3. Kaplan–Meier curves indicated that patients with high apo-C3 levels (> 8.5 mg/dL) had significantly lower freedom from MACEs than those with lower apo-C3 levels (≤ 8.5 mg/dL) (*P* < 0.001). (**D**) Kaplan–Meier curves between patients with high %NC and those with low %NC. Kaplan–Meier curves indicated that patients with high %NC (> median value = 16.675%) had significantly lower freedom from MACEs than did those with low %NC (≤ median value = 16.675%) (*P* < 0.001).

The levels of total cholesterol (TC), triglycerides (TG), non-high-density lipoprotein cholesterol (HDL-C), apo-A1, apo-B, and apo-C3 were higher in the high apo-C3 group than in the low apo-C3 group (Table 2). The LDL-C levels were lower in the high apo-C3 group than in the low apo-C3 group. Figure 1A shows the coronary angiography (CAG) findings in a representative case (a male patient in the high apo-C3 group, with an apo-C3 level of 8.8 mg/dL at enrollment) and VH-IVUS findings of the lesion. The intra- (r = 0.98 and 0.99) and inter-observer (r = 0.96 and 0.98) measurement variability were acceptable. CAG images revealed an intermediate lesion in the proximal right coronary artery (Fig. 1A a). Drug-eluting stents were implanted in the target severe stenotic tandem lesion in the left mid-circumflex coronary artery (mid-CX) (Fig. 1A b–d). The patient received statin treatment due to hyperlipidemia. The LDL-C level was 55 mg/dL 8 months later, and at that time, the patient had an acute myocardial infarction in the mid-right coronary artery (Fig. 1A e). The stenting left CX site showed no restenosis (Fig. 1A f). Figure 1A g (cross-sectional image) and h (longitudinal image) show the pre-PCI VH-IVUS findings of the target lesion of the mid-CX, which revealed thin-cap fibroatheroma with NC (red region) and dense calcium (DC) (white region). The %NC was 22.43%.

Comparisons of grayscale IVUS, VH-IVUS data, and MACEs between the low and high apo-C3 groups are presented in Table 3.

Grayscale IVUS data, including %plaque burden, were all comparable between the low and high apo-C3 groups. Regarding VH-IVUS data, the percentage of the fibrofatty (FF) volume in the plaque volume (%FF) was significantly lower in the high apo-C3 group than in the low apo-C3 group. Meanwhile, the %NC volume was significantly higher in the high apo-C3 group than in the low apo-C3 group. Total MACE and the incidence of new lesions were significantly higher in the high apo-C3 group than in the low apo-C3 group.

### Correlations between apo-C3 levels and four vessel compositions

Furthermore, we investigated the correlations between apo-C3 levels and four vessel compositions evaluated by VH-IVUS. Only the %NC among the four compositions significantly correlated with the apo-C3 level (R = 0.2109, *P* = 0.037) (Fig. 1B). Moreover, we evaluated the correlations of the four vessel compositions with each other. The percentage of the fibrous (FI) volume in the plaque volume (%FI) significantly inversely correlated with the %FF (R = -0.4628, *P* < 0.001) and percentage of the DC volume in the plaque volume (%DC) (R = -0.5343, *P* < 0.001). The %FF significantly inversely correlated with the %NC (R = -0.7520, *P* < 0.001) and %DC (R = -0.3868, *P* < 0.001). The %NC significantly positively correlated with the %DC (R = 0.5991, *P* < 0.001). A matrix showing the correlations between lipid levels, apolipoprotein levels, and plaque components is shown in Supplementary Table S2.

### Kaplan–Meier curves for comparisons between patients with high apo-C3 and those with low apo-C3

Kaplan–Meier curves (Fig. 1C) revealed that patients with apo-C3 levels > 8.5 mg/dL (high apo-C3; *n* = 46) had significantly lower freedom from MACEs than those with apo-C3 levels ≤ 8.5 mg/dL (low apo-C3; *n* = 52) (*P* = 0.0026). The mean survival time with high apo-C3 was 33.3 ± 2.7 (95% confidence interval [CI] 28.0–38.6) months, while that with low apo-C3 was 44.4 ± 1.4 (95% CI 41.7–47.1) months. Interestingly, patients with high %NC (> median value = 16.675%) of the lesion plaque showed significantly lower freedom from MACEs than those with low %NC (≤ median value = 16.675%) of the lesion plaque (Fig. 1D). The mean survival time with high %NC was 35.6 ± 2.5 (95% CI 30.6–40.5) months, while that with low %NC was 42.8 ± 1.8 (95% CI 39.2–46.3) months.

### Cox proportional hazards regression model for 4 year MACEs

In the univariate analysis, the %NC (hazard ratio [HR], 1.1207; 95% CI 1.0337–1.2151; *P* = 0.0057), %DC (HR, 1.1454; 95% CI 1.0482–1.2581; *P* = 0.0046), and high apo-C3 (HR, 3.4977; 95% CI 1.4696–8.3258; *P* = 0.0047) were significant risk factors for 4 year MACEs. Moreover, the multivariate analysis showed that the %NC (HR, 1.0960; 95% CI 1.0099–1.1894; *P* = 0.028) and high apo-C3 (HR, 2.9381; 95% CI: 1.2106–7.0820; *P* = 0.0171) were strong predictors of 4 year MACEs (Table 4). Additionally, a multivariate Cox hazards analysis adjusted for LDL-C and apo-B (model 1) showed that the %NC (HR, 1.1025; 95% CI 1.0157–1.1967; *P* = 0.0197) and high apo-C3 (HR, 2.6498; 95% CI 1.0465–6.7096; *P* = 0.0398) could predict 4 year MACEs. Similarly, in a model adjusted for apo-A1 and estimated glomerular filtration rate (eGFR) (model 2), %NC (HR, 1.1021; 95% CI 1.0127–1.1994; *P* = 0.0243) and high apo-C3 (HR, 2.9415; 95% CI 1.2163–7.1139; *P* = 0.0166) were independent predictors of 4 year MACEs (Table 5).

**Table 4:** Univariate and multivariate Cox hazards analyses for predicting 4 year MACEs.

Variable	Unadjusted HR	95% CI	*P*-value	Adjusted HR	95% CI	*P*-value
Male sex	2.0606	0.7092–5.9871	0.1840			
BMI	1.0813	0.9826–1.1900	0.1095			
Smoking	0.5491	0.2205–1.3674	0.1978			
Statin	1.9332	0.8871–4.2128	0.0972			
eGFR	0.9999	0.9472–1.0263	0.9958			
HDL-C	1.0190	0.9906–1.0481	0.1914			
LDL-C	0.9889	0.9760–1.0020	0.0974			
Apo-A1	1.0081	0.9889–1.0277	0.4114			
Apo-B	1.0111	0.9960–1.0262	0.1509			
Apo-C3	1.0758	0.9691–1.1943	0.1704			
%FI	0.9581	0.9028–1.0166	0.1571			
%FF	0.9509	0.8962–1.0089	0.0955			
%NC	1.1207	1.0337–1.2151	0.0057*	1.0960	1.0099–1.1894	0.0280*
%DC	1.1454	1.0482–1.2581	0.0046*			
High apo-C3	3.4977	1.4696–8.3258	0.0047*	2.9381	1.2106–7.0820	0.0171*

*apo-C3* Apolipoprotein C3; *MACE* Major cardiovascular event; *HR* Hazard ratio; *CI* Confidence interval; *BMI* Body mass index; eGFR, Estimated glomerular filtration rate; *HDL-C* High-density lipoprotein cholesterol;*LDL-C* Low-density lipoprotein cholesterol; *%FI* Percentage of the FI volume in the plaque volume; *%FF* Percentage of the FF volume in the plaque volume; *%NC* Percentage of the NC volume in the plaque volume; *%DC* Percentage of the DC volume in the plaque volume; high apo-C3, patients with apo-C3 > 8.5 mg/dL. * *P* < 0.05.

**Table 5:** Multivariate Cox hazards analyses of predictors of 4-year MACEs, adjusted for major confounders.

Variables	Model 1		Model 2	
	HR (95%CI)	P	HR (95%CI)	P
High apo-C3	2.6498 (1.0465–6.7096)	0.0398*	2.9415 (1.2163–7.1139)	0.0166*
%NC	1.1025 (1.0157–1.1967)	0.0197*	1.1021 (1.0127–1.1994)	0.0243*
LDL-C	1.0014 (0.9810–1.0163)	0.8968		
Apo-B	0.9883 (0.9611–1.0163)	0.4094		
Apo-A1			1.0076 (0.9887–1.0268)	0.4318
eGFR			1.0064 (0.9812–1.0322)	0.6242

*MACE* Major cardiovascular event; *HR* Hazard ratio; *CI* Confidence interval; *apo-C3* Apolipoprotein C3; *high apo-C3* Patients with apo-C3 > 8.5 mg/dL; *%NC* percentage of the plaque volume that is a necrotic core; *LDL-C* low-density lipoprotein cholesterol; *eGFR* estimated glomerular filtration rate. * *P* < 0.05.

### Comparison of VH-IVUS data between the new lesion plaque after PCI and the angiographical non-stenotic lesion plaque of his own pre-first PCI

We could estimate 5 new lesions in the high apo-C3 group by VH-IVUS at approximately 9–12 months after the first PCI. The %NC of the new lesion plaque was significantly higher than that of non-stenotic lesion plaque of his own pre-first PCI (Table 6).

**Table 6:** Comparison of VH-IVUS data between the new lesion plaque at 9–12 months after PCI and the angiographical non-stenotic lesion plaque of his own pre-first PCI in the high apo-C3 group (*n* = 5).

	Non-stenotic lesion plaque pre-first PCI	New lesion plaque after PCI	P
%Plaque burden	59.0 ± 3.4	67.2 ± 4.0	0.0362*
%FI	65.4 ± 1.7	56.4 ± 2.3	0.0819
%FF	17.7 ± 3.0	14.7 ± 2.2	0.4678
%NC	14.4 ± 1.1	20.6 ± 1.9	0.0236*
%DC	2.7 ± 0.5	8.3 ± 2.1	0.0628

Values are presented as mean ± standard errors. *P < 0.05 by paired t-test. Five new lesions in the high apo-C3 group could be estimated by VH-IVUS before PCI at 9–12 months after first PCI. VH-IVUS, virtual histology-intravascular ultrasound; *%FI* percentage of the FI volume in the plaque volume; *%FF* percentage of the FF volume in the plaque volume; *%NC* percentage of NC volume in the plaque volume ; *%DC* percentage of the DC volume in the plaque volume; high apo-C3, patients with apo-C3 > 8.5 mg/dL.

### Multivariate logistic regression analyses for 4 year MACEs

In the multivariate logistic regression analyses, high apo-C3 (OR, 3.2817; 95% CI 1.1321–9.5126; P = 0.0286) and %NC (OR, 1.1219; 95% CI 1.0030–1.2550; *P* = 0.0442) were independent predictors of 4 year MACEs after adjustment for LDL-C and apo-B (model 1). In model 2, adjusted for apo-A1 and eGFR, high apo-C3 (OR, 3.4861; 95% CI 1.2452–9.7600; *P* = 0.0174) was an independent predictor of 4-year MACEs (Table 7).

**Table 7:** Multivariate logistic regression analysis of predictors of 4-year MACEs, adjusted for major confounders.

Variables	Model 1		Model 2	
	OR (95% CI)	P	OR (95% CI)	P
High apo-C3	3.2817 (1.1321–9.5126)	0.0286*	3.4861 (1.2452–9.7600)	0.0174*
%NC	1.1219 (1.0030–1.2550)	0.0442*	1.149 (0.9973–1.2465)	0.0558
LDL-C	1.0036 (0.9775–1.0303)	0.7915		
Apo-B	0.9873 (0.9499–1.0186)	0.3548		
Apo-A1			1.0066 (0.9827–1.0310)	0.5910
eGFR			1.0029 (0.9710–1.0360)	0.8586

*MACE* Major cardiovascular event; *OR* odds ratio; *CI* confidence interval; *apo-C3* apolipoprotein C3; *high apo-C3* patients with apo-C3 > 8.5 mg/dL; %NC, percentage of plaque volume that is a necrotic core; *LDL-C* Low-density lipoprotein cholesterol; *eGFR* Estimated glomerular filtration rate. *The value of OR is statistically significant with *P* < 0.05.

### Oral medication before and after PCI

In order to determine their association with long-term prognosis, the oral medications used before and after PCI were compared (Supplementary Table S3). The results showed no significant differences. However, a comparison of medication uses between before and after PCI showed that antiplatelet drug use after PCI was more common than before PCI in the low apo-C3 group. There were no significant differences in other medications.

## Discussion

In this study, we clarified the relationship between apo-C3 and vessel composition in patients with SCD who underwent PCI. The %NC in the lesion plaque was higher in the high apo-C3 group than in the low apo-C3 group. Interestingly, the %NC significantly correlated with apo-C3. Moreover, the overall survival rate from MACEs was significantly lower in the high apo-C3 group than in the low apo-C3 group. Additionally, the overall survival rate from MACEs was significantly lower in the high %NC group than in the low %NC group. Furthermore, Cox hazards analysis proved that a high apo-C3 level and the %NC were independent predictors of 4 year MACEs, with the multivariate analysis also showing similar results. Our findings provide insights into the prognostic effect of apo-C3 and the %NC in the vessel components of patients with SCD after PCI, and the relationship between these factors was correlated. Therefore, apo-C3 may induce NC volume development and could be a useful predictor of MACEs after PCI in patients with SCD. To the best of our knowledge, this is the first study to show the contribution of the %NC to long-term MACEs in patients with SCD after PCI and the first study to demonstrate the correlation between apo-C3 and the %NC.

Apo-C3, an important regulator of lipoprotein metabolism, is strongly associated with hypertriglyceridemia and CVD progression. In addition to its effects on lipid metabolism, apo-C3 directly influences atherosclerosis development through several routes, such as facilitating the subendothelial accumulation of LDLs by increasing their affinity for artery wall proteoglycans^15–18^. The mechanism underlying this interaction is complex because apo-C3 does not bind to artery wall proteoglycans but appears to provoke changes in the lipid composition of lipoproteins, causing apo-B to adopt a conformation more favorable for proteoglycan binding^15,16^. Apo-C3 may also promote the aggregation and fusion of retained lipoproteins in the artery wall by activating sphingomyelinases^19,20^. Furthermore, apo-C3 facilitates the interaction between monocytes and endothelial cells, promotes smooth muscle cell proliferation, and induces inflammation by activating adhesion molecules and proinflammatory nuclear factor-κB in monocytes and endothelial cells^21,22^.

To date, several reports ^8–13^ have indicated that a high apo-C3 level is a predictor of future cardiovascular and ischemic cerebrovascular events in patients with CVD. Katzman et al.^13^ showed that apo-C3 concentrations in chylomicron-free serum were independently associated with event-free survival in patients with coronary artery disease in both fasting and postprandial states. However, patients in that study did not undergo PCI, and the authors did not look at vascular composition. In a study conducted by Sacks et al.^10^, patients experienced a recent myocardial infarction but did not undergo PCI. In their study, Scheffer et al.^9^ included Caucasian patients and excluded patients with type 2 diabetes mellitus who did not necessarily have coronary artery disease. In any case, previous studies have not always included all PCI patients. In our study, the significantly more common type of MACE was the occurrence of new lesions. In this sense, the presence or absence of PCI may not be relevant to the results; however, we are the first to demonstrate that higher apo-C3 is worse in the prognosis of PCI patients. In addition, no previous studies have examined the relationship between apo-C3 and vascular structure.

Apo-C3 did not affect the grayscale IVUS data (e.g., vessel volume and %plaque burden) but altered the proportion of the composition of blood vessels. Apo-C3 decreased the %FF and increased the %NC. The NC may be formed with a matrix-devoid gruel of lipids and cell debris absorbed by macrophages and dendritic cells in the intima in a process that remains incompletely understood in vivo^14^. A close interplay among dendritic cells and other immune cells results in full-blown immune activation, paving the way for a detrimental rupture of the atherosclerotic plaque^23^. A larger NC also confers a greater risk than a small NC ^14,24^. In our study, patients with high %NC had a worse prognosis than those with low %NC. Furthermore, the %NC was an independent predictor of 4-year MACEs after PCI in patients with SCD. Additionally, we have shown a significant positive correlation between apo-C3 and %NC values, which suggests that apo-C3 influences the development of the %NC. To the best of our knowledge, these results have not been reported before. Furthermore, %NC before PCI probably shows not only the predisposition of the existing plaques, but also the presence of plaque at risk in other coronary segments. In our study, 5 new lesions in patients in the high apo-C3 group that had progressed within the 9–12 months and had been examined using VH-IVUS at the follow-up examination, showed a significantly larger necrotic core in the new lesion than that of the angiographical non-stenotic lesion pre-first PCI. Considering this, the pre-PCI apo-C3 value may be able to predict the presence of predisposed plaques in other coronary segments that may progress in the near future.

Since the %NC cannot be assessed without VH-IVUS, which is an invasive method, it may be possible to predict future myocardial infarction if apo-C3, which can be assessed by blood sampling, is checked regularly as a surrogate marker in the outpatient clinic.

### Study limitations

This study has some limitations. First, this was a single-center study with a small sample; therefore, large-scale multicenter studies are needed to confirm our findings. However, with the advent of high-resolution IVUS, only VH-IVUS is used for clinical research purposes now, making it more difficult to conduct a study, since it is used less frequently. The possibility that unknown confounding factors might have influenced the results cannot be completely denied, even though multivariate analyses adjusted for known prognostic factors. However, there is no drug bias, as the oral medication was virtually unchanged during the study period. Furthermore, the correlation between apo-C3 and %NC volume of pre-PCI plaques is clear. The %NC volume of plaques with new lesions in the high apo-C3 group was as high as 20.6% at 9–12 months after the first PCI and was much higher than those of pre-PCI plaques. These results support the fact that high apo-C3 increases %NC and creates new lesions.

Second, because of the small sample size, we could not examine additional confounding factors for apo-C3. Finally, most of the new lesions were discovered on repeated CAG at 9–12 months after PCI. Nowadays, it is difficult to obtain informed consent to perform CAG 9–12 months after PCI. However, in routine clinical practice, new lesions cannot be traced.

In conclusion, apo-C3 facilitates NC progression in the coronary arteries of patients with SCD and increases the risk of future cardiovascular events. Apo-C3 may be a potent surrogate marker for predicting future cardiovascular events in patients with SCD after PCI. Apo-C3 levels should be monitored periodically in outpatient medical examinations. If apo-C3 level is high, closer examination using coronary computed tomography, stress test, or CAG should be performed to monitor CVD progression.

## Methods

This study was approved by the Ethics Committee of Fukushima Red Cross Hospital (Fukushima City, Japan; approval no. 2012–2) and performed in accordance with the Ethical Guidelines for Clinical Research of the Japanese Ministry of Health, Labour and Welfare. Informed consent was obtained from all individual participants included in the study.

### Study population

Our sample included a total of 98 consecutive patients with SCD who visited our hospital between November 1, 2012, and March 10, 2015, and underwent PCI with VH-IVUS. PCI was indicated in accordance with the Japanese consensus criteria^25^. After obtaining written informed consent, PCI and pre-IVUS were performed. All 98 patients were enrolled in this study, all of whom underwent coronary artery stenting in the target vessels. The degree of coronary artery stenosis was determined by consultation between two or more experienced interventional cardiologists (Y.S, K.S, A.I, F.H, and K.N). IVUS measurements were assessed by two investigators (TO and TS) who were blinded to the treatment allocation.

### IVUS image acquisition

IVUS examinations of the culprit lesions were conducted immediately before PCI. A phased-array, 20 MHz, 3.2-F IVUS catheter (Eagle Eye; Volcano Corp., Rancho Cordova, CA, USA) was placed in the distal coronary artery and pulled back to the aorto-ostial junction using a motorized catheter pull-back system set at 0.5 mm/s (Eagle Eye; Volcano Corp.). The grayscale IVUS and captured radiofrequency data were recorded by a DVD recorder.

### Grayscale and VH-IVUS analyses

Offline grayscale and VH-IVUS analyses were conducted using echo Plaque 4.0 software (INDEC Systems Inc., Los Altos, CA, USA). Corresponding proximal and distal reference IVUS images were identified for each culprit lesion and analyzed to determine the plaque volume within the involved arterial segment. Grayscale IVUS analysis was conducted using the American College of Cardiology Clinical Expert Consensus Document on Standards for Acquisition, Measurement, and Reporting of Intravascular Ultrasound Studies^26^. In the gray-scale conventional IVUS analysis, images were assessed to determine the lesion length, lumen volume, plaque volume, and vessel volume. In the VH-IVUS analysis, the FI, FF, NC, and DC regions were color coded. Estimates of each factor’s contribution to the volume of the entire culprit lesion plaque volume were reported as a percentage of the factor’s volume in the plaque volume of the culprit lesion (i.e., NC)^27^. The same investigators (TO and TS) subsequently reanalyzed the images with respect to the intra- and inter-observer reproducibility of the measurements.

### Clinical laboratory measurements

Laboratory evaluations of plasma lipid and apo levels were conducted within 2 days before PCI. Commercial reagent kits (Determiner L TC II, L LDL-C II, L HDL-C II, and L TG II; Kyowa Medex, Tokyo, Japan) were used to analyze total plasma cholesterol, LDL-C, HDL-C, and TG levels. Lipid concentrations were measured using an automated biochemical analyzer (Labospect 006; Hitachi, Tokyo, Japan). Apo-C3 was measured in chylomicron-free samples, and apo-B and a were measured using immunonephelometry. High-sensitivity C-reactive protein levels were measured using nephelometry. Apo levels were measured at a central clinical biochemistry laboratory (SRL Inc., Tokyo, Japan).

### Definition of 4 year MACEs

MACEs within 4 years (48 months) were defined as a composite of cardiac death, acute myocardial infarction, unstable angina, coronary revascularization for restenosis of a target vessel and new lesion, and endovascular therapy for arteriosclerosis obliterans. These events were ascertained by reviewing patients’ medical records and confirmed by direct dialog with patients, their families, and their physicians after discharge. If no events were experienced in about 8 months after PCI, the patients underwent repeated CAG at 9–12 months from first PCI. For > 90% stenosis in a target vessel (i.e., restenosis) or a new lesion, patients underwent revascularization using PCI; these events constituted MACEs.

### Statistical analyses

Data are presented as mean ± standard errors. Categorical and continuous variables of clinical characteristics were compared between the low and high apo-C3 groups, which were divided by the median apo-C3 value. Categorical data were analyzed using the chi-square test, and continuous data using Student’s *t*-test. All statistical analyses were two-sided. The significance level was set at *P* < 0.05. The comparisons of grayscale IVUS, VH-IVUS data, and MACEs between the low and high apo-C3 groups were performed in the same manner. In some patients, the comparisons of VH-IVUS data between the new lesion plaque at approximately 9–12 months after the first PCI and the plaque of his own which had angiographically non-significant stenosis at pre-first PCI were performed by paired *t*-test.

To allow for a more detailed analysis, the MACEs within 4 years-follow up were divided into whether they were new lesions (new plaque) or restenoses (same plaque); and characterized as requiring endovascular therapy or leading to AMI, UAP, or cardiac death. (Supplementary Table S1).

Pearson’s correlations between lipid levels, apolipoprotein levels, and plaque components (i.e., %FI, %FF, %NC, and %DC) were evaluated (Supplementary Table S2). Kaplan–Meier curve analysis was conducted, and the 48 month MACE-free survival was compared between the low and high apo-C3 groups, which were divided by the median apo-C3 value, using the log-rank test; and between the low and high %NC groups, which were divided by the median %NC value, using the Wilcoxon test.

A Cox proportional hazards regression model was used to estimate the long-term MACE HR and 95% CIs based on the univariate analysis of clinical, laboratory, and IVUS data and multivariate analysis of high apo-C3 levels adjusted for potential confounders (i.e., factors with *P* < 0.05 in the univariate analysis). Specifically, %NC and high apo-C3 were estimated by multivariable Cox proportional hazards regression models adjusted for major confounding variables such as LDL-C and apo-B (model 1), and apo-A1 and eGFR (model 2).

Similarly, multivariate logistic regression analyses of the association of high apo-C3 and %NC with MACEs within the 4 years following PCI were performed with adjustment for LDL-C and apo-B (model 1), and apo-A1 and eGFR (model 2).

All statistical analyses were performed using Ekuseru-Toukei for Windows version 1.02 (SSRI Co., Ltd., Tokyo, Japan).

## Supplementary Information

Supplementary Information.

## Data Availability

All data generated or analyzed during this study are included in this published article.
