# Supplementary material for: Apolipoprotein C3 and necrotic core volume are correlated but also associated with future cardiovascular events
Source: Sci Rep. 2022 Aug 25;12:14554. doi: 10.1038/s41598-022-18914-1 (PMC9458721; doi:10.1038/s41598-022-18914-1)
Supplement: Supplementary file 1 — Supplementary Information. [file 41598_2022_18914_MOESM1_ESM.docx]

**Supplementary Information**

**Supplementary Table S1.**

**Supplementary Table S2.**

**Supplementary Table S3.**

**Supplementary Table S1. MACEs within 4 years’ follow-up divided according to new lesion or restenosis**

|  | **New lesion (new plaque)** | | **Restenosis (same plaque)** | | **Total** |
| --- | --- | --- | --- | --- | --- |
|  | **High apo-C3**  **(Number of Re-PCI)** | **Low apoC3**  **(Number of Re-PCI)** | **High apo-C3**  **(Number of Re-PCI)** | **Low apo-C3**  **(Number of Re-PCI)** | **(Number of Re-PCI)** |
| **AMI** | **1 (1)** | **1 (1)** | **0** | **0** | **2 (2)** |
| **UAP** | **3 (3)** | **1 (1)** | **0** | **1 (1)** | **5 (5)** |
| **Effort AP** | **2 (2)** |  | **0** |  | **2 (2)** |
| **Silent ischemia** | **9 (6)** | **1 (1)** | **2 (1)** | **2 (2)** | **14 (10)** |
| **Cardiac death** | **1** |  | **0** |  | **1** |
| **EVT** | **2** |  | **0** |  | **2** |
| **Total** | **18 (12)** | **3 (3)** | **2 (1)** | **3 (3)** | **26 (19)** |

**MACE, major cardiovascular event; High apo-C3, patients with apo-C3 >8.5 mg/dL; Low apo-C3, patients with patients with apo-C3≤8.5 mg/dL; AMI, acute myocardial infarction; UAP, unstable angina pectoris; effort AP, effort angina pectoris; silent ischemia, significant coronary stenosis (>90%) with no symptoms; EVT, endovascular therapy.**

**Supplementary Table S2.Matrix of correlations between lipid levels, apolipoprotein levels and plaque components.**

**Table of correlation coefficients**

|  | TG | LDL-C | HDL-C | apo-B | apo-A1 | apo-C3 | %FI | %FF | %NC | %DC |
| --- | --- | --- | --- | --- | --- | --- | --- | --- | --- | --- |
| TG | 1.0000 | 0.1256 | -0.3724 | 0.5014 | -0.0812 | 0.5868 | 0.1507 | -0.0546 | 0.0212 | -0.1751 |
| LDL-C | 0.1256 | 1.0000 | 0.0329 | 0.8251 | 0.0288 | 0.0437 | 0.0117 | 0.0420 | -0.0290 | -0.0749 |
| HDL-C | -0.3724 | 0.0329 | 1.0000 | -0.1852 | 0.8384 | 0.0045 | -0.0866 | -0.0942 | 0.1206 | 0.2007 |
| apo-B | 0.5014 | 0.8251 | -0.1852 | 1.0000 | -0.0037 | 0.2534 | 0.0628 | 0.0103 | 0.0106 | -0.1489 |
| apo-A1 | -0.0812 | 0.0288 | 0.8384 | -0.0037 | 1.0000 | 0.1106 | -0.0745 | -0.0768 | 0.1112 | 0.1531 |
| apo-C3 | 0.5868 | 0.0437 | 0.0045 | 0.2534 | 0.1106 | 1.0000 | -0.0188 | -0.1164 | 0.2109 | 0.0070 |
| %FI | 0.1507 | 0.0117 | -0.0866 | 0.0628 | -0.0745 | -0.0188 | 1.0000 | -0.4628 | -0.1565 | -0.5343 |
| %FF | -0.0546 | 0.0420 | -0.0942 | 0.0103 | -0.0768 | -0.1164 | -0.4628 | 1.0000 | -0.7520 | -0.3868 |
| %NC | 0.0212 | -0.0290 | 0.1206 | 0.0106 | 0.1112 | 0.2109 | -0.1565 | -0.7520 | 1.0000 | 0.5991 |
| %DC | -0.1751 | -0.0749 | 0.2007 | -0.1489 | 0.1531 | 0.0070 | -0.5343 | -0.3868 | 0.5991 | 1.0000 |

**Test of uncorrelated mother correlation coefficients**

|  | TG | LDL-C | HDL-C | apo-B | apo-A1 | apo-C3 | %FI | %FF | %NC | %DC |
| --- | --- | --- | --- | --- | --- | --- | --- | --- | --- | --- |
| TG | - | 0.2202 | P < 0.001 | P < 0.001 | 0.4292 | P < 0.001 | 0.1406 | 0.5956 | 0.8369 | 0.0863 |
| LDL-C |  | - | 0.7476 | P < 0.001 | 0.7782 | 0.6694 | 0.9088 | 0.6810 | 0.7766 | 0.4634 |
| HDL-C | ** |  | - | 0.0680 | P < 0.001 | 0.9652 | 0.3965 | 0.3561 | 0.2368 | 0.0475 |
| apo-B | ** | ** |  | - | 0.9714 | 0.0118 | 0.5389 | 0.9198 | 0.9176 | 0.1433 |
| apo-A1 |  |  | ** |  | - | 0.2781 | 0.4661 | 0.4522 | 0.2756 | 0.1323 |
| apo-C3 | ** |  |  | * |  | - | 0.8544 | 0.2535 | 0.0371 | 0.9454 |
| %FI |  |  |  |  |  |  | - | P < 0.001 | 0.1239 | P < 0.001 |
| %FF |  |  |  |  |  |  | ** | - | P < 0.001 | P < 0.001 |
| %NC |  |  |  |  |  | * |  | ** | - | P < 0.001 |
| %DC |  |  | * |  |  |  | ** | ** | ** | - |

**Upper triangle: P-value/lower triangle: * P<0.05 , **P<0.01; TG, triglyceride; HDL-C, high-density lipoprotein cholesterol; LDL-C, low-density lipoprotein cholesterol; apo-B, apolipoprotein B; apo-A1, apolipoprotein A1; apo-C3, apolipoprotein C3; %FI, percentage of the fibrous volume in the plaque volume; %FF, percentage of fibrofatty volume in the plaque volume; %NC, percentage of necrotic core volume in the plaque volume; %DC, percentage of dense calcium in the plaque volume**

**Supplementary Table S3. The comparisons of oral medication between before and after PCI**

|  | **Low apo-C3 group (≤8.5 mg/dL)** | | | **High apo-C3 group (>8.5 mg/dL)** | | |  |
| --- | --- | --- | --- | --- | --- | --- | --- |
| **Oral medication** | **After PCI (n=47)** | **Before PCI (n=52)** | **P** | **After PCI**  **(n=45)** | **Before PCI (n=46)** | **P** | **P-after PCI** |
| **CCB** | **26 (55.3)** | **24 (46.2)** | **0.421** | **19 (43.2)** | **25 (54.3)** | **0.281** | **0.247** |
| **ACEI/ARB** | **21 (44.7)** | **25 (48.1)** | **1.000** | **21(46.7)** | **21 (45.7)** | **0.715** | **0.848** |
| **Antiplatelet drugs** | **42 (89.4)** | **38 (73.1)** | **0.028*** | **38 (84.4)** | **31 (67.4)** | **0.074** | **0.483** |
| **Anti-DM drugs** | **7 (15.2)** | **9 (17.3)** | **0.897** | **7 (15.4)** | **6 (13.0)** | **0.934** | **0.964** |
| **Fibrates** | **0 (0)** | **1 (1.9)** | **0.324** | **1 (2.2)** | **1 (2.2)** | **0.828** | **0.304** |
| **Statin** | **27 (57.4)** | **22 (42.3)** | **0.161** | **30 (66.7)** | **23 (50.0)** | **0.095** | **0.362** |

**There were 5 patients in the low apo-C3 group and 1 in the high apo-C3 group in whom the medication use after PCI could not be assessed.**

**Values are presented as means±standard errors or as numbers (percentages) of patients**

**P-after PCI, comparison between after PCI in the low apo-C3 group and after PCI in the high apo-C3 group; apo-C3, apolipoprotein C3; PCI, percutaneous coronary intervention; CAG, coronary angiography; CCB, calcium-channel blocker; ACEI/ARB, angiotensin-converting enzyme inhibitor/angiotensin II receptor blocker;**

**P-after PCI, * P<0.05**
